# Antiviral Action of Native and Methylated Lactoferrin and β-Lactoglobulin against Potato Virus Y (PVY) Infected into Potato Plants Grown in an Open Field

**DOI:** 10.3390/antibiotics9070430

**Published:** 2020-07-21

**Authors:** Mahmoud Sitohy, Soad Taha, Ali Osman, Mahmoud Abdel-Hamid, Ali Hamed, Ashraf Abdelbacki

**Affiliations:** 1 Department of Biochemistry, Faculty of Agriculture, Zagazig University, Zagazig 44511, Egypt; Aokhalil@zu.edu.eg; 2 Dairy Science Department, Faculty of Agriculture, Cairo University, Giza 12613, Egypt; soad_hassantaha@yahoo.com (S.T.); mahmoud.mohamed@agr.cu.edu.eg (M.A.-H.); 3 Virus and Mycoplasma Department, Agriculture Research Center, Giza 12619, Egypt; ali_hamed65@hotmail.com; 4 Plant Pathology Department, Faculty of Agriculture, Cairo University, Giza 12613, Egypt; amaeg@hotmail.com

**Keywords:** potato, PVY, esterification, tuber yield, dot blot hybridization, RT-PCR

## Abstract

Potato plants are liable to PVY infection without efficient control. Therefore, they were cultivated under greenhouse and open field conditions, artificially infected with PVY and then treated after 15 days of infection with native lactoferrin (LF) and native β-lactoglobulin (BL) and their esterified forms, MLF (methylated lactoferrin) and BLM (methylated β-lactoglobulin) to test the efficiency of this approach. Viral replication was inhibited by the applied substances, particularly the methylated forms, in a concentration-dependent manner, where the concentration of 500 μg·mL^−1^ was sufficient for plant protection against the PVY infection. An open field experiment showed that one single application of the antiviral substance was enough for maximum inhibitory action against PVY. The modified milk proteins induced higher inhibitory action on PVY virus replication in the plants, compared to their native forms, which was reflected by potato growth and yield. Using the dot blot hybridization and RT-PCR techniques to detect PVY in the experimental plants showed the supremacy of native and esterified LF in inhibiting the targeted virus. The generally observed scanning electronic microscopy (SEM) structural deformations and irregular appearance in PVY particles when treated with MLF and BLM revealed their direct action. BLM, MLF and LF are efficient antiviral agents against PVY. They can not only abolish the observed PVY-induced reduction in potato growth and tuber yield, but also further increase them to higher levels than negative control.

## 1. Introduction

Potato virus Y (PVY, species *Potato virus Y*, genus *Potyvirus*, family *Potyviridae*), is an ssRNA virus that is transmitted by numerous species of aphids [1]. PVY has a wide host range, and, although it is mainly limited to potato and tobacco, it infects other solanaceous crops [2,3]. PVY is widely considered as an important pathogen affecting the yield and quality of potato plants [4], representing a serious hindrance to the production and quality of seed potato. It has rapidly evolved in recent decades, becoming a complex of strains evading many farm managements practices. The evolution of PVY strains is continuously occurring, which is linked to several human influences, leading to the rapid change in PVY populations [5]. The buildup of virus levels in the vegetative propagated crop gradually weakens the seed sources and reduces plant vigor and tuber yields over several generations. Thus, the potato seed certification programs have monitored high virus loads every year, discarding huge seed lots with excessive amounts of virus, thus limiting the number of generations during which potatoes could be used for seed production [6]. Hence, PVY remains the major reason for the rejection of seed lots when exceeding the tolerance limits [7].

Breeding PVY resistant cultivars have long been an important trait in European breeding programs and is gaining traction in USA, but developing and accepting these new PVY resistant varieties will take a long time [5]. The continuous viral evolution may also be capable of escaping this specific resistance, and so other methodologies are still needed to manage PVY propagation. Aphids are mostly capable of transmitting plant viruses including PVY. Although mineral oils were reported to be successful in controlling aphids, managing aphids has only been partially successful in restricting PVY propagation [8].

Thus, other interventions may be sought for future use. Some natural proteins may have inherent antiviral activity and others may acquire this property by chemical modification [9,10]. Chemical modification of proteins by esterification has been proven to be a good tool; it incurs an enhanced net positive charge through neutralizing the free carboxyl groups of the side acidic amino acids [11,12,13,14,15,16,17]. This chemical change will enable the modified proteins to interact with many microorganisms including viruses [15,18,19,20,21,22,23]. The interaction between the esterified proteins and DNA was visualized *in vitro* through electrophoresis [24,25,26], by differential spectroscopy [27] and through affecting the PCR amplification of DNA [24,25]. Furthermore, the *in vivo* replication of M13 bacteriophage and lactococcal bacteriophages was evidently inhibited by the presence of esterified proteins [28,29]. Human and plant viruses were also found to be susceptible to esterified milk proteins [30,31,32,33,34,35,36]. The present study was designed to assess the potential antiviral inhibitory action of native and esterified milk proteins against PVY infection in potato plants in the field to establish a new approach for controlling this virus.

## 2. Results

**Figure 1:**
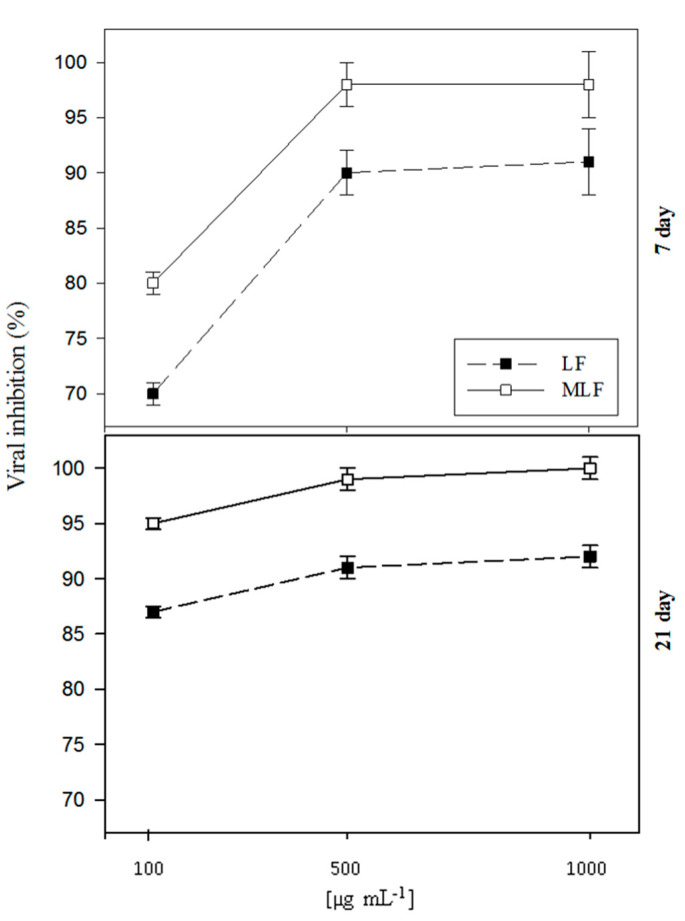
Inhibition of PVY replication into potato plants by foliar spray with lactoferrin (LF) and methylated lactoferrin (MLF) at three concentrations (100, 500 and 1000 μg·mL^−1^ distilled water). The viral load was determined in potato leaves after 7 and 21 days by the double antibody sandwich enzyme linked immunosorbent assay (DAS-ELISA) method. Six representative plant samples out of a total of 15 plants/treatment were used in this analysis and the results were expressed as the means ± SE.

The data in Figure 1, derived from a preliminary experiment conducted under greenhouse conditions, delineate the antiviral activity of native and methylated lactoferrin (LF and MLF) against PVY infection in potato plants.

Viral inhibition was calculated according to the following equation:

Viral inhibition %=[(Cp−Cn)−(T−Cn)/(Cp−Cn)]×100

where *Cp* is the positive control reading, *Cn* = the negative control reading and *T* is the treatment reading.

The substances were applied as a foliar spray at three different concentrations (100, 500 and 1000 µg mL^−1^) and the virus load was determined by the DAS-ELISA technique (double antibody sandwich enzyme-linked immunosorbent assay) in the leaves of the treated plants after 7 and 21 days of the treatment. The treatments inhibited viral replication in a concentration-dependent manner. This trend was particularly evident when the measurements were made after 7 days of the treatment. It can also be observed that there was no significant difference between the two higher concentrations (500 and 1000 µg mL^−1^) either after 7 or 21 days. However, a considerable difference was seen between the native and modified forms at the time points of examination. It can be concluded that the concentration of 500 µg mL^−1^ is enough for protecting against PVY viral infection. The level of viral inhibition continued after extending the time of examination to 21 days after treatment, showing the stability of the antiviral action.

**Figure 2:**
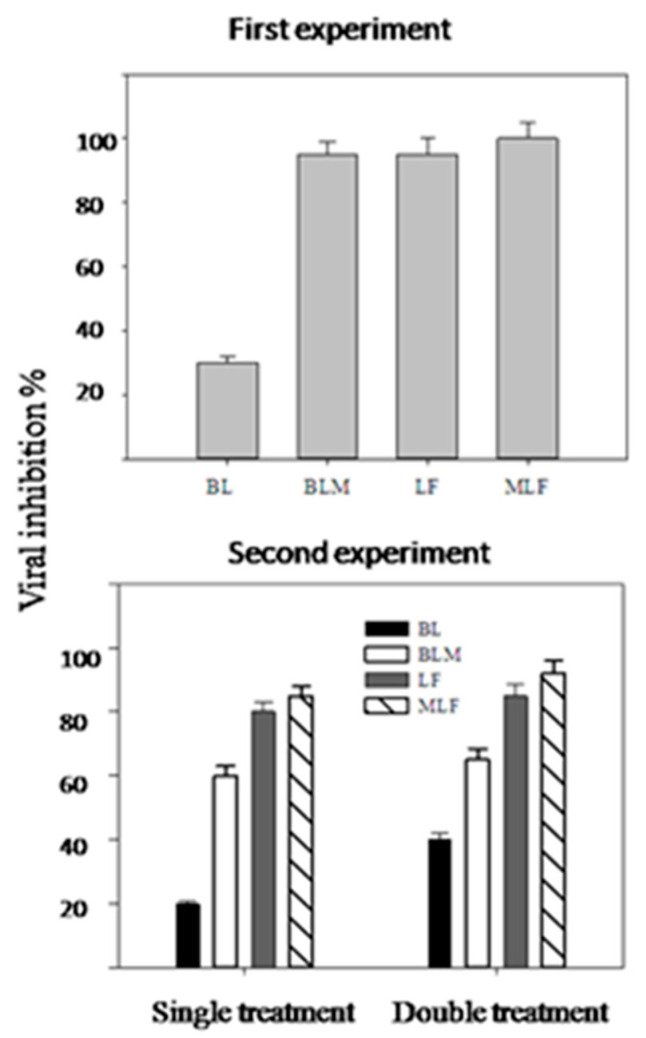
Inhibition of PVY replication in potato plants by application of different milk proteins at a single treatment of 500 µg mL^−1^ distilled water after 15 days of infection. The antiviral action was measured after 21 days of treatment by double antibody sandwich enzyme linked immunosorbent assay (DAS-ELISA) (First experiment). The second experiment followed the design of the first experiment except that plants were sprayed either once (after 10 days) or twice, after 10 and 20 days of viral infection, and the plant samples were assayed for virus load after 21 and 31 days of the first spray. Six representative plant samples out of a total of 15 plants/treatment were used in this analysis and the results are expressed as the means ± SE. Viral inhibition is calculated according to the following equation:

The antiviral potentiality of BL (β-lactoglobulin), BLM (methylated β-lactoglobulin), LF and MLF, applied as a single foliar spray at 500 μg mL^−1^ against PVY, was studied. The results in Figure 2 (upper graph) clearly indicated that all protein treatments could inhibit the PVY replication to different degrees. Modified proteins (BLM and MLF) showed higher inhibitory action than their corresponding native forms (BL and LF), i.e., 94 and 100% against 30 and 94%, respectively. We also noticed that there were large differences between the two native forms, as LF (94%) was much stronger than BL (30%). Complete inhibition of viral propagation was observed with MLF against the PVY.

Viral inhibition of PVY by single or double application (500 µg mL^−1^) of the tested substances (applied 10 and 20 days after infection) was measured and the results are shown in Figure 2 (lower graph). The data herein indicate that the modified proteins have considerably higher inhibitory effects on the PVY replication than native proteins except for LF, where the difference between the modified and the native was relatively low. For the modified milk proteins (BLM, MLF), or even the native LF, there were no big differences between the double or single treatment.

**Figure 3:**
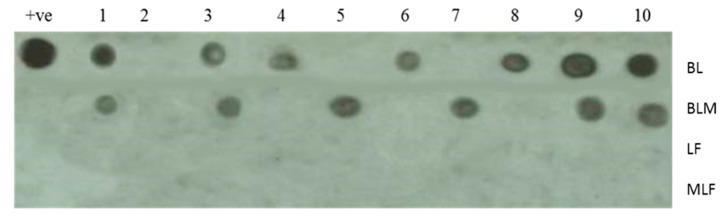
Detection of PVY in the leaves of treated potato plants using dot blot hybridization technique in the leaves of potato cultivated under open field conditions and treated once with 500 µg mL^−1^ (15 days after the viral infection) of BL and LF or BLM and MLF. The numbers from 1 to 10 refer to different potato plant samples of the same treatment and + ve is the positive control. The negative control was run separately and did not show any spot (data not shown). Ten representative plant samples out of a total of 15 plants/treatment were randomly selected and used in this analysis.

Figure 3 demonstrates virus detection in potato plant leaves by dot blot hybridization in 10 plants of each treatment taken at random 31 days after viral infection. It is seen that in potato plants treated with native BL, the number of plants positive for the virus detection were 7, i.e., 70% of plants were hosting the viral infection. For the plants treated with the BLM, the number of plants positive for the viral detection was reduced to 60%. In plants treated with LF and MLF, the virus was not detected in any treated plant sample.

Based on the dot blot hybridization technique results, it seems that both native and modified proteins have antiviral action against the replication of the PVY in potato plants. For β-lactoglubulin, esterification has augmented the antiviral action against PVY but there was no difference between native and esterified forms of LF. The native LF is already a basic protein and thus has the potentiality of viral interaction as previously reported [37].

Viral inhibition %=[(Cp−Cn)−(T−Cn)/(Cp−Cn)]×100

where *Cp* is the positive control reading, *Cn* = the negative control reading and *T* is the treatment reading.

**Figure 4:**
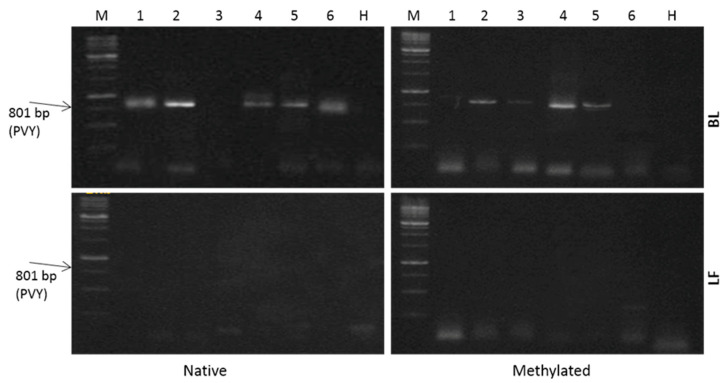
Detection of PVY in potato leaves using one step RT-PCR guided by Verso ^TM^ one step RT- PCR kit (Thermo scientific) and visualized by gel electrophoresis in the leaves of potato plants cultivated under open field conditions receiving a single application of 500 µg mL^−1^ of LF and BL, or their methylated forms, (MLF and BLM, respectively). Six representative plant samples out of a total of 15 plants/treatment were used in this analysis.

Using RT-PCR, the data in Figure 4 confirm the data obtained by DAS-ELISA and dot-blot hybridization that the modified proteins generally have enhanced antiviral action against potato virus PVY, more than their native forms, except LF, which is highly active in its native form. Both LF and MLF achieved 100% viral elimination, against 60% in the case of BLM.

**Figure 5:**
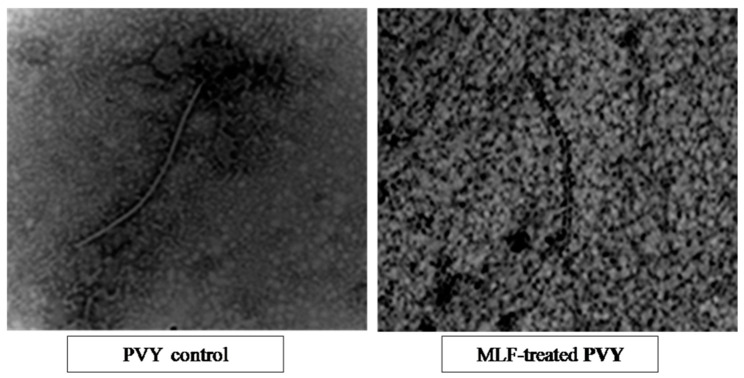
Scanning electron microscopy (SEM) images of potato virus PVY in its native form (control) and after being in contact with 500 µg mL^−1^ BLM and methylated lactoferrin (MLF) at room temperature for 1 h.

The images of scanning electronic microscopy (SEM) in Figure 5 (60,000×) show that the presence of MLF in the medium of the virus-infected plant cells could protect them against viral action. The viral morphological appearance of the untreated virus shows a regular filamentous viral particle, while the one treated with MLF seems rather cracked and irregular. The SEM of the BLM-treated virus is quite similar to the MLF-treated virus (image not is shown).

The influence of BL, BLM, LF and MLF (500 µg mL^−1^) on the yield traits of potato plants infected with PVY are shown in Table 1. The plants infected with PVY showed a 19.2% reduction in the total yield of potato tubers/feddan. Treatment with the modified proteins has not only prevented the yield loss caused by the virus but shown relative increases compared to the negative control. BLM and MLF incurred additional yield increases over the negative control amounting to 18 and 13%, respectively. The relative increases in potato tuber yield compared to the positive control were 48% and 42% in the case of BLM and MLF, respectively. Other yield traits (fresh, dry weight, plant height, no. of shoots, no. of tubers and weight of tubers/plant) were also promoted by the action of the modified milk proteins. The dry weight of the whole plant was also clearly influenced by all treatments. The viral infection reduced the dry weight by about 6% compared to the negative control. Treating the infected plants with the native protein forms (BL and LF) enhanced the plant dry weight by about 13 and 14% over the negative control, respectively. Moreover, treating the infected plants with the modified protein forms (BLM and MLF) increased their dry weight by about 32 and 38% over the negative control, respectively.

## 3. Discussion

The esterification extent of both BLM and MLF was 100% and the isoelectric point was 9.5 and 10.5, against 4.5 and 8.5 for BL and LF, respectively (data not shown). These high iso-electric points (IEPs) as well as the increased hydrophobicity incurred by esterification, may explain their bio-reactivity against the virus.

The data derived from the preliminary experiment suggest the presence of a proportional relation between the tested substance concentration and its antiviral action against PVY. LF is a previously reported antiviral [37], whose increased antiviral activity by esterification is due to an increase in both the net positive charge and the hydrophobicity [15,17,22,38]. It could be concluded that the concentration of 500 µg mL^−1^ is sufficient for protecting plants against PVY infection. The small difference (10%) between the native and modified LF in the antiviral action, indicates the original high activity of native LF. The absence of important differences between the viral load after 7 and 21 days of treatment proves the stability of the antiviral action of this substance, at least for 21 days.

The two open field experiments showed that the modified milk proteins (BLM, MLF) had higher inhibitory action on virus propagation in plants compared to their native forms, probably due to the augmented positive charges incurred by the esterification reaction. The lower difference between native and modified LF is probably due to their close IEPs (9.5 and 10.5, respectively as compared to the large difference between BL and BLM (4.5 and 8.5, respectively). The relatively higher antiviral action of MLF compared to BLM may also originate from its higher IEP (10.5 against 8.5, respectively).

The dot blot hybridization technique has also confirmed the supremacy of native and esterified LF in inhibiting PVY and the superiority of BLM over BL as a result of the esterification process. This technique indicated the absence of PVY in plants treated with either LF or MLF as the native form is already a basic protein and has the potential to interact with the virus [37,39].

RT-PCR confirmed the results obtained by dot blot hybridization. Although this technique could spot the difference between the modified protein BLM and BL, it could not detect any difference between MLF and LF, since the latter is originally highly active [39].

The SEM images of PVY hosted in potato plants leaves, subjected to MLF, showing evident structural deformations and irregular appearance, confirm the direct action of the substance on the viral particles. The similarity between the SEM images of the BLM-virus and MLF-virus indicates a similar mechanism of interaction and effect. There is a possibility that the virus outer structure has been disrupted through protein–protein interactions, i.e., the viral coat protein and the antiviral protein probably via electrostatic interactions or hydrophobic aggregation supported by MLF hydrophobicity augmented by the esterification process. LF was previously elucidated to interact with DNA and RNA molecules [40] via some potential binding sites. Due to its high positive charge, LF is able to bind a large number of compounds [41] and RNA and DNA viruses [42,43]. Moreover, LF was found to inhibit the entry of viral particles into host cells, by direct attachment to the viral particles or by blocking their cellular receptors [44]. BLM was also found to interact with nucleic acids [24,25,45], thus explaining the deformed appearance of the viral particles.

The PVY-infected plants showed markedly reduced plant and tuber weight in accordance with previous work [5]. The antiviral activities of BL, LF, BLM and MLF against PVY have been evidently reflected on the growth and yield of PVY-infected potato plants, particularly when treated with the methylated forms (BLM and MLF at 500 g. µg mL^−1^). The pattern of the plant dry weight increases in a similar trend to that found with the antiviral activity, i.e., the more antiviral active substance was administered, the more plant growth (expressed as dry weight). The apparent association between the antiviral activity and the plant dry weight shows the importance of plant antiviral agents in protecting plant growth and maintaining normal yield. Moreover, treating the PVY-infected plants with BLM and MLF did not only abolish the virus-incurred tuber yield reduction but further increased it to levels higher than the negative control (19 and 13% increases, respectively). Surprisingly, although BLM was less antiviral than MLF, the rate of yield increases by the two substances (BLM and MLF) were at the same level. This may nominate BLM as an antiviral agent of equal potency to MLF. The increases in both dry weight and tuber yield to levels not only superior to the infected plants (viral control), but also to levels higher than the negative control, may refer to a general growth-stimulating effect of the used substances, either native or modified. The superiority of action of the modified, compared to the native, proteins may be due to enhanced potential against the viral infection and propagation. This additional effect of the two proteins may agree with [46], who reported that LF potentially protects the host from viral infections through not only inhibiting virus attachment to the host cells, virus replication and enhancing the systemic immune functions of the host but also through promoting the regenerative properties of the host tissue [47]. BLM may mimic MLF and LF based on its high positive net charge. The obtained results agree with the study of [9], where BLM and MLF can stimulate tobacco seedlings against infection with tobacco mosaic virus (TMV) as a result of activated expression of a number of defense-related enzymes.

The superiority of action of the modified, compared to the native, proteins may also be due to enhanced potential against the viral infection and propagation incurred by the intensified positive charge, enabling them to interact and complex with the viral particles. This is in complete accordance with [10], who showed the superior action of the modified over the native LF, and found a secondary action of both forms in activating the expression of a number of defense genes. The observed success of the antiviral action of the substance by application on the plant surface by foliar spray agrees with previous studies using foliar spray of protein hydrolysates as bio-fertilizers [48], and also with studies that applied some proteins on the surface of some fruits during postharvest as antifungal agents [11,49]. However, further detailed studies might be needed to understand the different potential interactive pathways of the antiviral substance through its contact with host plant and the invading virus.

## 4. Materials and Methods

### 4.1. Tested Proteins

Proteins, LF and BL were esterified with methanol according to the procedure of [45,50], and the esterification extent was quantified by the color reaction with hydroxylamine hydrochloride [51]. The resultant modified proteins were denoted as BLM and MLF.

### 4.2. Virus and Plants

White burley tobacco plants infected by PVY were collected from the greenhouse farm based on the visible viral infection symptoms. The infected leaves were crushed in distilled water and squeezed through double layer muslin cloth. The filtrate was centrifuged at 5000× *g* for 10 min and the supernatant was used as the virus inoculum. The identity of the virus was verified and confirmed by electron microscopy and bio-molecular studies (RT-PCR and DAS-ELISA). PVY viruses in Egypt were found to exist in at least five distinct recombinant PVY strains including PVY^NTN^ and PVY^N-Wi^ [52]. The strain of the PVY used in this study was not specifically identified.

### 4.3. Preliminary Greenhouse Experiment

Potato plants (*Solanum tuberosum* cv. Spunta) were planted under greenhouse conditions, taking into consideration all the environmental requirements, e.g., irrigation and fertilization. The same potato cultivar was used in all the conducted experiments. To simulate the Egyptian conditions, potato plants need temperatures of 15–25 °C. At the beginning, the potato plant needs a relatively long day and a relatively short day later, during the formation of tubers. The soil acidity was in a moderate range (pH 5.2–5.5). The plants were subjected to artificial virus infection with PVY after 30 days of plantation. The cotyledonary leaves and the first leaf of potato plants (*Solanum tuberosum* cv. Spunta) were dusted with carborundum and inoculated with PVY virus (100 µL/leaf), and then washed with distilled water. The plants were treated with foliar spray of chemically modified proteins at different concentrations (0.0, 100, 500 and 1000 µg. mL^−1^ distilled water) after 15 days of infection. Samples were collected from leaves after 7 and 21 days of treatment and viral RNA was estimated by molecular hybridization analysis.

### 4.4. First Field Experiment

Potato plants (*Solanum tuberosum* cv. Spunta) were planted in the Faculty of Agriculture farm -Cairo University, taking into consideration all the ordinary environmental requirements, and infected with PVY as previously mentioned. The experiment followed a randomized complete block design in a factorial arrangement using three replicates (5 plants each), totaling 15 plants per treatment. Each treatment included 15 plants. The experiment included six treatments; negative control, positive control and PVY- infected plants treated with either BL, LF, BLM or MLF. Negative control plants (healthy plants) were inoculated with distilled water only. Positive control plants were inoculated by PVY only. The plants were treated once by the tested substances (500 µg mL^−1^ distilled water) after 15 days of infection. Samples were collected from leaves after 21 days from treatment for detection of PVY by DAS-ELISA using polyclonal antiserum from rabbit (LOEWE^®^Biochemica GmbH, Sauerlach, Germany), electron microscopy, RT-PCR and dot blot hybridization [31].

### 4.5. Second Field Experiment

This experiment was designed following the system of the first experiment to confirm the previous results and to verify whether we needed to treat infected potato plants (*Solanum tuberosum* cv. Spunta) with the modified proteins once or twice to completely inhibit virus propagation. Therefore, the implementation of this experiment was as in the first experiment except that potato plants were treated twice, after 15 and 25 days of infection by the tested proteins (500 µg mL^−1^ distilled water). Samples were analyzed for the virus after 21 days from the last treatment.

### 4.6. Final Field Experiment

The design of the first experiment was followed and potato plants (*Solanum tuberosum* cv. Spunta) were grown to the harvesting stage for two successive seasons to estimate fresh weight (g), dry weight (g), the number of shoots, plant height (cm), number of tubers/plant and weight of tubers/plant (g) and to estimate the total yield. The yield increase (%) was calculated relative to negative control (*NC*) as in the following equation:

%Increase=[(weight of treatment−weight of NC)/weight of NC]×100

The increase in yield relative to the positive control (*PC*) was calculated as:

%Increase=[(weight of treatment−weight of PC)/weight ofPC]×100

### 4.7. PVY Detection

PVY was detected in the samples of the first experiment and confirmed in the final experiment by DAS-ELISA using polyclonal antiserum from rabbit, electron microscopy, RT-PCR and dot blot hybridization. In the second experiment, only DAS-ELISA analysis was used.

#### 4.7.1. Double Antibody Sandwich Enzyme Linked Immunosorbent Assay (DAS-ELISA) Assay

The DAS-ELISA assay was used according to [53] to estimate the relative viral load in potato leaves after different treatments in the first and second experiment. After mixing the leaf extracts with the polyclonal antisera from rabbit (LOEWE^®^Biochemica GmbH, Sauerlach, Germany), the developed color was measured at 405 nm. This technique was used to conclude the viral inhibition in the treated plants relative to the positive control and the negative control according to the following equation:

Viral inhibition %=[(Cp−Cn)−(T−Cn)/(Cp−Cn)]×100

where *Cp* is the positive control reading, *Cn* = the negative control reading and *T* is the treatment reading.

#### 4.7.2. RT-PCR Detection of PVY

Potato leaves representing six different treatments (the experiment included six treatments; negative control, positive control and PVY- infected plants treated with either BL, LF, BLM or MLF) were tested for the PVY infection using RT-PCR and six potato plants from each treatment (15 plants) were tested. Total RNA was isolated from potato leaves using gene jet™ RNA purification kit (Fermentas, Waltham, MA, USA) according to the instruction manual. One-step RT-PCR was performed using Verso^TM^ one-step RT- PCR kit (Thermo scientific, Waltham, MA, USA). RT-PCR mix (25 µL) included: 3 µL RNA (4 ng/µL), 12.5 µL of one step PCR master mix (2x), 3 µL 10 µM of each primer, 0.5 µL Verso enzyme mix, 1.25 µL RT-Enhancer and 4.75 µL of nuclease-free water. The operating PCR program consisted of 35 cycles of (1 min at 94 °C, 1 min at 55 °C and 2 min at 72 °C). The following specific primers were used:

PVYCPvBamH1: TCAAGGATCCGCAAATGACACAATTGATGCAGG
PVYCPcEcoR1: AGAGAGAATTCATCACATGTTCTTGACTCC

The RT-PCR products were stained with gel star (Lonza, USA) and analyzed on 1% agarose gels in 0.5X TBE buffer then visualized by UV illumination using the Gel Documentation System (Gel Doc ± 00, Bio-Rad, Hercules, CA, USA). The expected size for the RT-PCR product of the positive samples was 801 pb.

#### 4.7.3. Dot Blot Hybridization Test

Non-radioactive hybridization was used to detect presence of PVY virus in the tested plants and to evaluate the antiviral activity of tested proteins. Five microliters of extracted PVY-RNA (PCR product) samples were dot onto the positively charged nylon membrane. The specific probe was prepared as described in [54]. The hybridization experiments were carried out using Gene Images AlkPhos and Chemiluminescent Detection System signal generation and detection with CDP-Star (Amersham, Biosciences, UK Limited, Uppsala, Sweden) [31,54].

#### 4.7.4. Scanning Electron Microscopy (SEM)

Scanning electron microscopy (JEOL-SEM, Tokyo, Japan) was used to evaluate the antiviral activity of tested proteins as described in [55].

### 4.8. Statistical Analysis

The data was analyzed by using SPSS 24.0 for Windows (SPSS Inc., Chicago, IL, USA) and expressed as the mean ± standard deviation (SD). The variation was assessed by one-way (ANOVA) and the differences between experimental groups were calculated by Duncan’s multiple-range test [56].

## 5. Conclusions

The observed correlation between the concentration and the antiviral action of the tested substances against PVY confirms BLM, MLF and LF as PVY-antivirals. The concentration 500 µg mL^−1^ is sufficient to control virus infection as a single application with antiviral action lasting at least 21 days after the treatment. The modified milk proteins (BLM, MLF) have higher inhibitory action on PVY virus replication than their native forms probably due to their higher positive charges incurred by the esterification reaction. The relatively higher antiviral action of MLF than BLM may also originate from the alkaline nature of the original LF, which was further enhanced by esterification.

The studied substances may counteract viral infection and propagation through their direct action on the virus particles as deduced from the observed SEM structural deformations and irregular appearance of PVY particles treated with the antivirals. Alternatively, the studied substances can hinder the viral replication in the plant cells via the electrostatic and hydrophobic reactions between the positive charges and hydrophobic regions on the esterified protein and the negative phosphate backbone side and hydrophobic nitrogen base side of viral RNA exposed during the replication process.

The markedly reduced tuber growth and yield by PVY-infection can be abolished by treating plants with the methylated proteins (BLM or MLF). The consistency between rate of plant growth and yield with the antiviral activity confirms the protective role of the substance. Treating the PVY-infected plants with BLM and MLF did not only abolish the virus-negative effects but further enhanced plant performance to levels higher than the negative control. This action may refer to other metabolic ameliorating pathways, as BLM was less antiviral than MLF. Still, the rate of yield increase by both substances were significantly in the same level.

## Figures and Tables

**Table 1:** Effect of antiviral BLBLM, LF and MLF (500 µg mL^−1^) on the yield traits of potato plants infected with potato virus Y (PVY). Viral artificial infection was conducted 30 days after plantation and the foliar spray of the treating substance was applied 15 days after the viral infection.

Parameters	NC *	PC **	BL	BLM	LF	MLF
No. of shoots	3.10 ± 0.14 a	4.4 ± 0.14 d	3.4 ± 0.14 b	3.8 ± 0.14 c	3.15 ± 0.21a	3.8 ± 0.14 c
Plant height (cm)	58 ± 0.07 d	52.55 ± 0.78 a	54.8 ± 1.13 c	54.95 ± 0.64 c	53.75 ± 1.06 b	53 ± 0.57 b
No of tubers/plant	6.05 ± 0.07 a	6.265 ± 0.09 b	6.515 ± 0.16 c	6.985 ± 0.12 e	6.2 ± 0.14 b	6.79 ± 0.08 d
Fresh weight (g)	382 ± 2.83 b	348.5 ± 2.12 a	356 ± 4.24 c	456.5 ± 3.54 c	356.5 ± 2.12 c	441.5 ± 2.12 d
Dry weight (g)	60.5 ± 0.78 b	56.75 ± 1.06 a	67.365 ± 1.9 c	79.4 ± 1.98 d	68.45 ± 2.05 c	83.7 ± 0.99 e
Tubers/plant (g)	396.50 ± 2.1b	346.5 ± 3.54 a	433 ± 1.41 c	516 ± 1.41 e	431.5 ± 2.12 c	494 ± 2.83 d
Total yield (ton/feddan)	8.27 ± 0.09 b	6.6 ± 0.14 a	8.53 ± 0.18 c	9.79 ± 0.13 de	8.155 ± 0.08 b	9.36 ± 0.08 d
% Increase/NC	-	-	3	18	−1	13
% Increase/PC	-	-	29	48	23	42

Data are the means of two successive seasons ± SE (15 plants/treatment for each season). Different letters in the same row indicate significant difference * Negative control (received neither viral infection nor treatment), ** Positive control (received viral infection but not treatment). The data of all 15 plants/treatment were used in this analysis and the results are expressed as the means ± SE. Different letters in the same row indicate significance at *p* < 0.05.
